# Alcohol Use Unmasking Heterozygous Hereditary Hemochromatosis

**DOI:** 10.7759/cureus.52364

**Published:** 2024-01-16

**Authors:** Serra Sozen, Akash Shah

**Affiliations:** 1 Medicine, University of Vermont, Burlington, USA; 2 Primary Care, Danbury Hospital, Danbury, USA; 3 Internal Medicine, Nuvance Health, Brookfield, USA

**Keywords:** iron homeostasis, iron storage diseases, iron overload, hereditary hemochromatosis, alcohol use disorder

## Abstract

Hereditary hemochromatosis (HH) is an autosomal recessive disorder characterized by excess iron absorption in the body following a mutation in the HFE gene. Though prolonged iron deposition has been shown to cause clinical symptoms such as hyperpigmentation, arthralgias, and liver damage, many individuals remain asymptomatic and exhibit no signs of iron overload. Here, we present a case where a 34-year-old with a history of severe alcohol use disorder presented with high iron, ferritin and transferrin saturation levels indicative of iron overload. Further testing for HFE gene mutations revealed simple heterozygote C282Y status, confirming the diagnosis of hereditary hemochromatosis. Simple heterozygotes, however, typically do not present with any symptoms of iron overload. This patient was counseled on lifestyle modifications which included abstaining from alcohol and reducing iron and vitamin C intake. As a result, his iron panel parameters improved. Thus, our case highlights that excessive alcohol consumption can exacerbate hereditary hemochromatosis and risk for overload even among heterozygotes.

## Introduction

Hereditary hemochromatosis (HH) is a genetic disorder of autosomal recessive inheritance that is characterized by the progressive accumulation of excess iron in the body, leading to structural and functional abnormalities in organs such as the pancreas, heart and notably liver [1]. Iron overload is typically identified by derangements in serum iron, transferrin saturation and serum ferritin levels, in addition to clinical symptoms that range from skin bronzing, hepatomegaly, restrictive cardiomyopathy and generalized weakness [2]. HH is easily treated with regular therapeutic phlebotomy until iron levels are stable. Early intervention is typically crucial, as HH can predispose to irreversible organ damage, such as cirrhosis, hepatocellular carcinoma or heart failure [3]. 

HH is coded for by the human homeostatic iron regulator (HFE) gene on chromosome 6, which encodes the transmembrane glycoprotein HFE that is responsible for modulating iron absorption. In individuals with hemochromatosis, the HFE gene contains a base substitution mutation of tyrosine for cysteine at amino acid 282 (C282Y), and is oftentimes present in a homozygous state in over 60% of the American population with HH. In addition to the C282Y mutation, other gene mutations have been identified in less severe forms of HH, namely the H63D and S65C variant alleles [4]. Interestingly, compound heterozygotes - carriers of both the HFE C282Y and H63D mutations - have been found to be at increased risk of hereditary hemochromatosis and associated iron overload, though the penetrance of this genotype is quite low [5]. Simple heterozygotes - carriers of just C282Y - however, often do not develop iron overload or organ damage [6]. It is also important to note that phenotypic expression of hereditary hemochromatosis can heavily be influenced by environmental factors, such as diet, sex, or frequency of treatment within the context of other comorbidities [7]. 

The association between alcohol consumption and iron overload specifically has been well established, given the synergistic hepatotoxic effects of each substance [8]. Iron and alcohol both cause release of reactive oxygen species and pro-inflammatory cytokines, leading to liver damage [9]. Furthermore, it has been postulated that alcohol has a direct effect on iron storage in the body. Alcohol-induced damage to hepatocytes may cause iron leakage into circulation. Alcohol consumption also impacts iron transfer from the intestine - thus changes in intestinal permeability may contribute to increased iron levels within circulation [10]. In-vitro and in-vivo research have additionally demonstrated that alcohol has been shown to have a direct down-regulatory effect on hepcidin expression, thereby inhibiting iron clearance [11]. One study found that a two-fold increase in intestinal iron absorption was observed among individuals with chronic alcohol use compared to controls who did not consume alcohol [10]. In this report, we present a case where a male C282Y simple heterozygote with alcoholism developed high iron, ferritin, and transferrin saturation indicating iron overload, in spite of this patient's young age and benign genotype. 

## Case presentation

A 34-year-old Caucasian male patient presented to his primary care outpatient office for follow-up following an emergency department visit for an acute gouty attack in the right metatarsophalangeal joint. Past medical history was significant for hypogonadotropic hypogonadism, reactive airway disease, anxiety, insomnia, gastroesophageal reflux disease, and alcohol use disorder. In the weeks prior to office presentation, this patient endorsed significant weight gain and abdominal bloating secondary to increased liquor consumption. Patient explained that he had been feeling especially anxious due to relationship and home stressors and was consuming up to half a bottle of vodka a day for several months. He expressed desire to quit alcohol and began attending individual and group therapy sessions, though he had never enrolled in a rehabilitation program or taken medications for alcohol use disorder. Patient's only home medications were colchicine for gout flare and escitalopram for anxiety. On office presentation, patient’s labs (Table 1) revealed abnormalities consistent with increased alcohol consumption, such as an elevated gamma-glutamyl transpeptidase (GGT) level of 285 U/L and an elevated aspartate aminotransferase (AST) level of 56 U/L. Labs also showed evidence of a B12 deficiency with a hemoglobin level of 14.5 g/dL and mean corpuscular volume (MCV) of 101 fl. This patient has chronically had elevated MCV seen on past labs, ranging from 101 to 107 fl. B12 level on this visit was measured at <150 pg/mL. Thus, he was started on B complex multivitamin. Further testing was performed to assess for presence of Intrinsic Factor blocking antibody, however this yielded negative results. This patient was also discovered to have elevated iron levels of 274 mcg/dL and elevated ferritin of 747 ng/mL with a transferrin saturation at 76%. Though serum ferritin is widely understood as an acute phase reactant and could be increased within the context of gout attack or fatty liver secondary to alcoholism, the additionally elevated iron level prompted suspicion for hemochromatosis or polycythemia. Of note, though the patient has never donated blood before, his condition has never been detected nor has he ever had tests performed to assess iron storage levels. Patient was counseled on abstaining from alcohol and advised to avoid any iron-containing supplements and multivitamins. On follow-up labs one month later, and approximately one month following alcohol cessation, this patient’s ferritin level had improved significantly at 409 ng/mL, which supported reactive etiology. Iron levels, total iron binding capacity (TIBC) and transferrin saturation returned at 152 mcg/dL, 356 mcg/dL and 43% respectively, along with normalized liver enzymes. An HFE DNA analysis was performed and found heterozygosity for C282Y mutation and the normal allele, as well as negativity for the H63D and S65C mutations.

**Table 1:** Laboratory Testing WBC white blood cells, RBC red blood cells, Tsat transferrin saturation, TIBC total iron binding capacity, MCV mean corpuscular volume, MCH mean corpuscular hemoglobin, MCHC mean corpuscular hemoglobin concentration, RDW CV red cell distribution width, AST aspartate aminotransferase, ALT alanine aminotransferase, ALKP alkaline phosphatase, GGT gamma-glutamyl transpeptidase

Laboratory Test	On Presentation	Repeat labs after one month	Reference ranges
WBC	8.5	7.7	4.5–10.0 10 × 3/μL
RBC	4.18	4.39	4.00–5.00 10 × 6/μL
Hemoglobin	14.5	14.6	12.0–16.0 g/dL
Hematocrit	42.2	43.5	42.0–51.0%
Iron	274	152	50-175 mcg/dL
Ferritin	747	409	12-300 ng/mL
Tsat	76	43	15%-50%
TIBC	359	356	240-450 mcg/dL
Vitamin B12	<150	NA	230-950 pg/mL
MCV	101	99	80–99 fl
MCH	34.7	33.3	27–35 pg
MCHC	34.4	33.6	32–36 g/dL
RDW CV	13.2	12.5	11.5–15.4%
Total bilirubin	0.8	0.5	0.2–1.3 mg/dL
AST	56	40	10–50 U/L
ALT	42	76	12–78 U/L
ALKP	110	108	46–128 U/L
GGT	285	NA	5–40 U/L
C282Y Hemochromatosis	NA	Heterozygous ABN	NA
H63D Hemochromatosis	NA	Negative	NA
S65C Hemochromatosis	NA	Negative	NA

## Discussion

This case report highlights the impact of alcohol consumption on the expression of hereditary hemochromatosis among heterozygotes. We find that increased alcohol intake can increase the severity of disease expression, demonstrated through higher iron parameters. Interestingly, this patient was not symptomatic. His presentation can be explained by his simple heterozygote phenotypic profile, as it is well-understood that C282Y heterozygotes are typically not at risk of developing symptomatic iron overload [7]. Several studies have suggested that alcohol may lower the iron threshold required to produce symptoms and cause liver damage [4,7]. Given the patient's young age, no history of hemolytic disorders or blood transfusions, and his benign HH genotype, we point to alcohol abuse as the most likely exacerbating factor for his iron overload. The marked decrease in his iron parameters following alcohol cessation also confirms this. However, other factors like this patient’s sex or diet could have played a role in lowering the threshold as well [9]. Of note, this patient was diagnosed with hypogonadotropic hypogonadism 3 years prior, specific symptoms of which were low testosterone levels, gynecomastia and decreased libido. Links between HH and hypogonadism of testicular or central origin have been described in various studies [12,13]. This patient did not have any abnormalities in testicular appearance or impairment of ejaculation, and labs indicated inappropriately normal levels of luteinizing hormone (LH) and follicle stimulating hormone (FSH). MRI of the pituitary was unremarkable. Still, this patient's symptoms of gonadal dysfunction could very well have been secondary to his previously undetected HH. It is also crucial to note that patients with hemochromatosis who consume alcohol risk developing more serious comorbidities. The additive effects of iron and alcohol on stellate cell activation and fibrogenesis have been associated with an increased risk of hepatic fibrosis, cirrhosis, malignancy and end-stage liver disease [14]. The condition of hereditary hemochromatosis must therefore be managed through preventative lifestyle changes such as abstinence from alcohol and reduced iron intake, with recommendation for regular phlebotomy if symptoms are present [15]. Future studies may benefit from investigating factors beyond alcohol in the exacerbation of hemochromatosis-related liver disease, such as infectious hepatitis or unhealthy dietary habits.

## Conclusions

Our study highlights how alcohol use can cause significant increases in iron storage parameters amongst individuals with heterozygous hemochromatosis. We further emphasize the importance of genetic testing for HFE mutations amongst patients with a history of excessive alcohol consumption, even if they present asymptomatically. Treatment should focus on controlling total body iron by avoiding risk factors that may promote iron uptake and by undergoing therapeutic phlebotomy when necessary. Further research will be needed to investigate the impact of alcohol on the progression of liver disease in simple heterozygotes with hereditary hemochromatosis.
